# Influence of play on positive psychological development in emerging adulthood: A serial mediation model

**DOI:** 10.3389/fpsyg.2022.1057557

**Published:** 2022-12-06

**Authors:** Wing W. Y. Ho

**Affiliations:** School of Education and Languages, Hong Kong Metropolitan University, Hong Kong, Hong Kong SAR, China

**Keywords:** play, emerging adulthood, emotional intelligence, resilience, positive emotions, serial mediation, broaden-and-build theory

## Abstract

**Introduction:**

Although the literature reports that play provides substantial psychological benefits for individuals, it is often only emphasized by educators for younger children but less so for emerging adults. This cross-sectional study examined the impact of time spent engaged in play on emerging adults’ emotional intelligence, emotional traits, and resilience.

**Methods:**

Participants (*N* = 131, 93.9% women) were full-time undergraduate students between the ages of 19 and 25 (*M* = 21.28, *SD* = 1.08).

**Results:**

Results showed that play experiences cultivated emotional intelligence and strengthened resilience. Further, the findings supported a serial mediation model in which play was indirectly related to resilience through its effect on emotional intelligence and positive emotion.

**Discussion:**

Play is recognized as a means to relieve stress and protect psychological wellbeing. Emerging adults who experience pleasure, enjoyment, learning, and the acquisition of new skills will benefit from play. Individuals should enhance positive play experiences, particularly sporting activities, to maintain a healthy work-life balance given the positive relationship between play, emotional intelligence, emotional traits, and resilience.

## Introduction

Emerging adulthood, a term initially proposed by Arnett (2000), is the distinct period between adolescence and young adulthood, that is, between the ages of 18 and 25. Literature suggest that emerging adulthood is a period of positive development (O'Connor et al., 2016; Lo-oh, 2019). Emerging adults, also known as “Generation Me,” have increasingly been described as narcissistic, self-absorbed, and unhappy (Twenge, 2014). The stereotypical emerging adult has difficulty entering the adult world (Padilla-Walker and Nelson, 2017). Additionally, this role change affects emerging adults, who are often exposed to an array of stressors and increased personal responsibility associated with a more complex social world.

Emerging adulthood is an emotionally complex time during which individuals develop careers and niches in society, enter adult relationships, and encounter potentially high levels of stress due to family, financial, and career obligations (Levinson, 1986; Huang et al., 2021; Fenzel and Richardson, 2022) and often experience emotional turmoil (Feldt et al., 2021). Hence, emerging adults should be helped to express more positive affect and vent negative emotions properly to increase their ability to manage life’s challenges. Emotional development is critical in emerging adults, as it promotes the experience, expression, understanding, and regulation of emotions from birth, as well as the evolution of these capacities at different life stages. Emotional intelligence refers to the individual’s ability to monitor and distinguish their own and others’ feelings and emotions, as well as the capacity to use this information to manage their relationships and guide their thinking and actions (Goleman, 1995; Mayer and Salovey, 1997). According to Salovey and Mayer’s (1990) framework of emotional intelligence, it is crucial for individuals to understand their positive and negative emotions, process emotional information accurately, and have the insight to skillfully use their emotions when solving problems and making plans. Tugade and Fredrickson (2004) supported that positive emotions are significantly related to each of these aspects.

Play is not trivial; it is essential to development. Play includes experiencing pleasure and enjoyment, learning, and acquiring new skills. It fosters psychological well-being, which affects resilience (Denovan and Macaskill, 2017). Numerous studies have confirmed that children perform important tasks during play, and, in turn, experience physical, intellectual, social, and emotional growth (Conner, 2007; Ginsburg et al., 2007). Play provides individuals with a diverse environment to express and explore their feelings, including fears, dreams, and disappointments. Individuals who frequently engage in age-appropriate play have better emotional competence (Bergin and Bergin, 2015). Play contributes to the holistic health and wellbeing of individuals at each stage of their lifespan; however, its role has not yet been fully realized (Bayliss and Etchells, 2016).

A literature review showed that little is known about the impact of play in emerging adulthood, and that the unanticipated consequences of reducing the psychological benefits of play are seldom studied. In particular, studies that relate emotional intelligence, emotional traits, and resilience to individual play experiences are scarce. Thus, the present study explored the relationship between play and its psychological benefits in emerging adults. More specifically, this study explored the impact of play on resilience using emotional intelligence and emotional traits as mediating variables in emerging adulthood.

### Psychological benefits of play and resilience during emerging adulthood

Although people know what play is, it can be difficult to define, owing to its complexity in behavior and context (Cooney, 2004). Consequently, the benefits of play are sometimes overlooked. While no consensus on the definition of play exists, some criteria are commonly identified in its depiction. For example, play is intrinsic, spontaneous, repetitive, pleasurable, and voluntary (Payne and Isaacs, 2020). Theoretically, play can be characterized into two major areas: the science of play, which recognizes play as a crucial part of human development that deserves serious study, and the art of play, in which the therapist and the child both participate and where there is joy, pleasure, and freedom (Tanta and Knox, 2015; Zaharia et al., 2022). Schaefer and Drewes (2013) identified 20 core agents of change in play therapy that can improve individuals’ attachment formation, self-expression, emotion regulation, resiliency, and stress management. The healing power of play can stem from its different aspects, such as frequency, type, length, the process of learning, and appropriateness of the content (e.g., Kearney et al., 2017; Yogman et al., 2018). By engaging in play, individuals may discover new ways of utilizing resources, getting support, or using coping strategies, and consequently learn to function effectively even in stressful situations (Mannello et al., 2020).

Resilience refers to a person’s innate ability to successfully cope with change or adversity (Lantieri and Goleman, 2014). Luthar (2006) posited that resilience is a construct that consists of two distinct dimensions: significant adversity and positive adaptation (Luthar et al., 2000). Hu and Gan (2008) defined resilience as a coping process by which individuals experience healthy and constructive adjustment after undergoing some form of severe adversity. Highly resilient people have a greater capacity to learn from setbacks and apply that knowledge to difficult life situations (Salovey et al., 1999; Tugade and Fredrickson, 2004). Thus, it is particularly important to understand resilience during emerging adulthood, as its associated changes in functional capacity have an important impact on life course outcomes (Wood et al., 2018).

The dynamic process of resilience involves an interplay between risk factors internal and external protective factors which can modify the impact of an adverse life event (Rutter, 2012). Furthermore, psychological resilience and negative emotions seem to be regulated by age, with the association stronger in adults than in children or adolescents (Hu et al., 2015), thus indicating that the resilience of emerging adults needs to be addressed. In designing interventions to promote resilience in emerging adults, a better understanding of their malleable and adaptive resources might be helpful (Luecken and Gress, 2010). Researchers have found a strong link between play and resilience development (Aris and Murgatroyd, 2017). Evidence has shown that individuals may develop through play the physical and emotional resilience necessary to overcome any survival challenge (Schaefer and Drews, 2013; Gray, 2019).

Fredrickson’s (2001) broaden-and-build theory suggests that positive emotions broaden people’s momentary thought-action repertoires and build their enduring personal resources. Thus, individuals who have more positive emotions are better equipped to overcome challenges by developing enduring physical, intellectual, psychological, and social resources (Fredrickson, 2001). However, as indicated previously, the relations of play and emotional development during emerging adulthood have rarely been studied. Therefore, the present study analyzed two emotional constructs (namely, emotional intelligence and emotional traits) as potential mediators in the relationship between play and resilience.

### Emotional intelligence and positive emotions as mediators

Emotional intelligence is “the capacity to reason about emotions, and of emotions to enhance thinking” (Mayer et al., 2004, p.197). A classic view of emotional intelligence is the four-branch model, as conceptualized by Mayer et al. (2004): (1) perception of emotions, (2) use of emotions, (3) understanding of emotions, and (4) managing emotions (Mayer et al., 2004; Sarrionandia et al., 2018). Thus, emotional intelligence reflects the ability to manage emotions effectively, productively, and adaptively (Sekhri et al., 2017). Emotional intelligence is an important internal protective factor (Pollard et al., 1999) and serves as an antecedent to resilience (Matthews et al., 2002), rather than encompassing resilience (Magnano et al., 2016). Emotional intelligence may be directly associated with resilience; for instance, it is easier for more emotionally intelligent individuals to adapt to stressful situations (Armstrong et al., 2011).

Emotional intelligence is assumed to buffer the impact of challenging situations through emotional self-awareness, expression, and management (Armstrong et al., 2011). There is considerable evidence showing its positive role in facilitating stress coping, building interpersonal relationships, and managing life’s transitions. Accordingly, emotional intelligence helps individuals to withstand adversity and rebound after encountering difficulties. Research has shown that emotional intelligence and resilience are enhanced through play (e.g., Lester and Russell, 2008; Macintyre, 2012). Play has been recognized as an ideal intervention for almost every conceivable problem (Hellendoorn et al., 1994). Although individuals develop a skill foundation through their experiences (e.g., unstructured play and interacting emotionally with others) in early childhood, these skills continue to be learned and developed later in life (Allen, 2018).

Emotions can be divided into two broad classes: positive and negative. Positive affect reflects a positive mood experience (e.g., happiness), while negative affect represents a negative mood experience (e.g., sadness and fear). Individuals experience different levels of positive and negative emotions while under stress (Folkman, 2008). In addition to eliciting positive emotions, trait emotional intelligence contributes to down-regulating negative emotions (Farnia et al., 2018). A study found that people who are resilient enjoy life and approach it with optimism, zest, and energy, are curious and open to new experiences, and are characterized by high positive emotionality (Block and Kremen, 1996).

The broaden-and-build theory of positive emotions (Fredrickson, 1998, 2000, 2001) provides a framework for understanding psychological resilience. Positive emotions build resilience (Cohn et al., 2009) and help buffer stress (Folkman and Moskowitz, 2000). However, negative emotions do not affect the benefits of positive emotions. Waugh (2014) stated that “the presence of positive emotions in resilient people does not necessarily coincide with fewer negative emotions” (p. 73). Studies have shown that positive and negative emotions can co-occur during stressful situations (Folkman and Moskowitz, 2000; Ong et al., 2006; Scott et al., 2014), and that this co-occurrence may be affected by the ability of resilient people to flexibly adapt their emotional responses to match the demands of environmental circumstances (Waugh et al., 2011). Individuals who experience relatively fewer positive emotions have lower resilience (Tugade and Fredrickson, 2004). In turn, individuals who cope more effectively with hardship, adversity, or trauma develop the ability to efficiently regulate their emotions. Thus, it seems that positive emotionality is an important element of psychological resilience. Further, research has indicated that positive affect can promote resilience through automatic activation of positive emotions when encountering stressful situations (Tugade and Fredrickson, 2008; Sewart et al., 2019). A study showed that highly resilient people proactively cultivated their positive emotionality by strategically eliciting positive emotions through play (Takiguchi et al., 2022). Hence, this study postulated that among the two predicted mediators in the relationship of play and resilience, emotional intelligence would precede positive emotions.

### The present study

As noted above, research has indicated that play positively impacts individuals’ psychological development. However, the impact of play on emerging adults has rarely been investigated. In light of this, this study aimed to explore the following hypotheses:

*H1:* Emerging adults’ play experiences positively predict resilience (H1).

*H2:* Emerging adults’ play experiences positively predict emotional intelligence (H2a) and positive emotions (H2b).

*H3:* Emotional intelligence plays an intermediary role in the relationship between play experiences and resilience in emerging adults (H3).

*H4:* Positive emotions play an intermediary role in the relationship between the play experiences and resilience in emerging adults (H4).

*H5:* Emotional intelligence and positive emotions operate as serial mediators between emerging adults’ play experiences and resilience (H5).

## Materials and method

### Design

This cross-sectional study used surveys to identify factors related to the influence of play on the psychological development of emerging adults.

### Participants

The participants (*N =* 131, 93.9% women) were full-time undergraduate students at a Hong Kong university, studying early childhood education. Their ages ranged from 19 to 25 (*M* = 21.28, *SD* = 1.08). According to the student records, all participants undertaking this program were aged 25 years and younger. Although seven respondents did not indicate their age, their responses were included in the data set. The demographic characteristics of the study participants are presented in Table 1.

**Table 1:** Demographic characteristics of participants (*N* = 131).

	*n*	Percentage (%)
**Sex**		
Male	8	6.1
Female	123	93.9
**Age**		
19	1	0.8
20	20	15.3
21	50	38.2
22	34	26.0
23	12	9.2
24	5	3.8
25	2	1.5
Other^#^	7	5.3
**No. of family members**		
Two	10	7.6
Three	25	19.1
Four	53	40.5
Five	30	22.9
Six	13	9.9
**Family income**		
$5,001 – $10,000	5	3.8
$10,001 – $20,000	25	19.1
$20,001 – $30,000	14	10.7
$30,001 – $50,000	27	20.6
$50,001 or above	5	3.8
Do not Know	54	41.2
No Income	1	0.8

^#^Seven respondents did not indicate their age. Percentages have been rounded to one decimal place and as such may add up to slightly more than 100%.

### Measures

#### Perspectives of play experience

Henricks (2015) proposed six perspectives for viewing play: affect, action, interaction, activity, disposition, and context. These perspectives were incorporated into a multiple-choice questionnaire to explore the respondents’ play experiences over the last 12 months. The questions addressed types of play (Table 2), time spent on play/extracurricular activities (Table 3), and playmates (Table 4).

**Table 2:** Types of play/extracurricular activities in last 12 months (*N* = 131).

	*n*	Percentage (%)
**Participated in play/extracurricular activities**		
Yes	107	81.7
No	24	18.3
**Sports (football, basketball, cycling, archery, climbing, fencing, dancing, track and field, snooker, tennis, table tennis, badminton, yoga/aerial yoga, swimming, hiking, canoeing, running, squash, roller skating, volleyball, gym, cardio, wake surf, athletic cheerleading, rope skipping, figure skating, boxing, touch rugby, scooter)**		
Yes	100	76.3
No	31	23.7
**Music (classical piano/piano/electronic piano, violin, viola, cello, flute, saxophone, clarinet, guitar)**		
Yes	57	43.5
No	74	56.5
**Chess (go, Chinese chess, chess)**		
Yes	10	7.6
No	121	92.4
**Creative arts (watercolor painting, oil painting, sketch, pastel nagomi art, electronic art, handicrafts)**		
Yes	64	48.9
No	67	51.1
**Leisure activities (group game, watching TV, leisure reading, mobile game, TV game, video game, online game, board game)**		
Yes	129	98.5
No	2	1.5

**Table 3:** Time spent on play/extracurricular activities per week in last 12 months (*N* = 131).

	*n*	Percentage (%)
**Time spent on play/extracurricular activities per week**		
None	17	13.0
1 h or less	15	11.5
1 h	18	13.7
2 h	17	13.0
3 h	10	7.6
4 h	11	8.4
5 h	7	5.3
6 h	10	7.6
7 h	4	3.1
8 h	5	3.8
9 h	3	2.3
10 h	5	3.8
13 h	1	0.8
20 h	2	1.5
21 h	5	3.8
22 h or more	1	0.8
**Time spent on leisure reading per week**		
None	26	19.8
1 h or less	42	32.1
1 h	17	13.0
2 h	13	9.9
3 h	9	6.9
4 h	5	3.8
5 h	8	6.1
6 h	2	1.5
7 h	1	0.8
8 h	1	0.8
9 h	2	1.5
10 h	4	3.1
21 h	1	0.8
**Time spent playing video games per week (e.g., computer, mobile phone, PS5, Wii U, Xbox, I-pad)**		
None	2	1.5
1 h or less	2	1.5
1 h	6	4.6
2 h	12	9.2
3 h	12	9.2
4 h	7	5.3
5 h	4	3.1
6 h	8	6.1
7 h	4	3.1
8 h	10	7.6
9 h	3	2.3
10 h	9	6.9
11 h	1	0.8
12 h	6	4.6
13 h	3	2.3
14 h	4	3.1
15 h	7	5.3
16 h	1	0.8
17 h	1	0.8
18 h	5	3.8
20 h	3	2.3
21 h	8	6.1
22 h or more	8	6.1
Others^#^	5	3.8

^#^Three students spent 28, 35, and 50 h, respectively. Two students did not indicate the time spent. Percentages have been rounded to one decimal place and as such may add up to slightly more than 100%.

**Table 4:** Playmates in last 12 months (*N* = 131).

	*n*	Percentage (%)
**No playmates**		
Yes	10	7.6
No	121	92.4
**Siblings**		
Yes	60	45.8
No	71	54.2
**Parent**		
Yes	32	24.4
No	99	75.6
**Classmates**		
Yes	50	38.2
No	81	61.8
**Friends**		
Yes	105	80.2
No	26	19.8
**Neighbors**		
Yes	4	3.1
No	127	96.9
**Domestic Helpers**		
Yes	1	0.8
No	130	99.2
**Grandparents**		
Yes	3	2.3
No	128	97.7
**Others**		
Partner	1	0.8
Boyfriend	1	0.8
Family members	1	0.8
Relative	1	0.8
No	127	96.9

Percentages have been rounded to one decimal place and as such may add up to slightly more than 100%.

The experience of play comprises three dimensions, including enjoyment (e.g., “Did you enjoy the process of play?”), learning (e.g., “Did you learn new knowledge while you were playing?”), and new skills (e.g., “Did you learn new skills while you were playing?”). The items are measured using a five-point Likert-type scale ranging from 1 = “*totally disagree*” to 5 = “*totally agree*.” This scale measures the experience of play based on prior research (Smith and Vollstedt, 1985; Baboo et al., 2022). Higher scores indicate higher levels of positive play experience. In the current study, Cronbach’s alpha was 0.77 for the total scale.

#### Wong and Law emotional intelligence scale

The Wong and Law Emotional Intelligence Scale (WLEIS; Wong and Law, 2002) is a 16-item self-report scale grounded in the ability emotional intelligence theory. The WLEIS measures ability emotional intelligence (Boburka, 2020) based on Mayer et al.’s (2004) four-branch model and has four distinct dimensions: self-emotion appraisal (e.g., I have a good understanding of my own emotions), appraisal of others’ emotion (e.g., I am a good observer of others’ emotions), use of emotion (e.g., I am a self-motivated person), and regulation of emotion (e.g., I have good control of my own emotions). The items are measured using a five-point Likert-type scale that ranges from 1 = “*totally disagree*” to 5 = “*totally agree*.” Higher scores indicate higher levels of emotional intelligence. In the current study, Cronbach’s alpha was.88 for the total scale and ranged from.82 to.94 for the subscales.

#### Questionnaire of emotional traits

The Questionnaire of Emotional Traits (QET; Chang and Yeh, 2003) is a 32-item self-report questionnaire that measures respondents’ emotional traits across four subscales: happiness (e.g., I am always satisfied with my life), sadness and fear (e.g., I always feel sad), anger and disgust (e.g., I always feel angry), and desire (e.g., I always feel a sense of accomplishment). The items are measured using a four-point Likert-type scale, ranging from 1 = “*definitely do not feel*” to 4 = “*definitely feel*.” The sadness and fear and anger and disgust subscales are reverse scored and summed with the other subscale scores. Higher scores indicate more positive affect. In the current study, Cronbach’s alpha was 0.95 for the total scale and ranged from 0.78 to 0.91 for the subscales.

#### Resilience scale for Chinese adolescents

The Resilience Scale for Chinese Adolescents (RSCA; Hu and Gan, 2008) comprises 27 items across five different factors: affect control (e.g., I am always discouraged by failure), goal planning (e.g., I have specific goals in my life), help-seeking (e.g., When I’m in trouble and need help, I do not know who to turn to), family support (e.g., My parents always encourage me to do my best), and positive thinking in adversity (e.g., Compared to the result, the process is more beneficial to one’s growth). Items are scored using a five-point Likert-type scale, ranging from “1 = *completely disagree*” to “5 = *completely agree*.” Total scores represent the level of resilience, with higher scores representing higher resilience. This measurement was judged to be culturally appropriate for a Chinese sample. In the current study, Cronbach’s alpha was 0.81 for the total scale and ranged from 0.70 to 0.81 for the subscales.

### Procedure

The Ethical Principles of Psychologists and Code of Conduct of the American Psychological Association were adhered to. The study was reviewed and approved by the university’s research ethics committee. The anonymity and confidentiality of the data were ensured. Participants were selected using convenience sampling. Invitations were distributed *via* email to 500 university students. The email included an invitation letter and a link for students to click on if they agreed to participate in the online survey. Informed consent was required prior to participation. The introductory page assured the participants of anonymity, that their responses would be confidential, and that there was no foreseen physical and psychological harm. Additionally, respondents were informed that their participation was voluntarily that they could withdraw from the study at any stage without consequences.

### Data analyses

For descriptive statistical analysis, SPSS 28.0 was used to determine the means and standard deviations of time spent playing, play experiences, play habits, types of play, emotional variables, and participants’ characteristics. A frequency analysis was performed to describe the demographic information and the distribution of age and sex. Inferential statistics were utilized to make judgments on the probability of an observed difference or relationship. Harman’s single-factor test was used to test the majority of the variance, which can be explained by a single factor (McFarlin and Sweeney, 1992). The majority of variance was 22.42%. It can be assumed that no threat of common method bias was present (Podsakoff et al., 2003).

Pearson’s product–moment correlation analysis was to determine the relation between the variables. To test hypotheses 1 to 5, the SPSS PROCESS macro, Model 6 (Version 4.1; Hayes, 2022), and the bootstrapping method (Bollen and Stine, 1990) were used for serial mediation analysis, applying two significant mediators for each analysis. In the analyses, 95% confidence intervals and a 5% significance level were used to determine statistical significance (Cohen et al., 2016). Other than age, there were no missing data.

## Results

### Correlation analysis of play and the outcome variables

#### The psychological benefits of play

Correlations between experiences of play and the outcome variables are presented in Table 5. All variables were significantly correlated. Experiences of play had a weak, positive, and statistically significant correlation with emotional intelligence (*r* = 0.36, *p* < 0.001), emotional traits (*r* = 0.19, *p* < 0.05), and resilience (*r* = 0.35, *p* < 0.001). Emotional intelligence had a moderate, positive, and statistically significant correlation with emotional traits (*r* = 0.48, *p* < 0.001) and resilience (*r* = 0.58, *p* < 0.001). Emotional traits had a strong, positive, statistically significant correlation with resilience (*r* = 0.69, *p* <0.001).

**Table 5:** Means, standard deviations, and correlations among the variables.

Variable	*M*	*SD*	1	2	3	4
1. Play	11.38	2.02	-			
2. Emotional intelligence (WLEIS)	55.79	8.17	0.36^***^	-		
3. Emotional traits (QET)	81.72	14.95	0.19^*^	0.48^***^	-	
4. Resilience (RSCA)	84.45	10.74	0.35^***^	0.58^***^	0.69^***^	-

*N* = 131. Emotional Intelligence: WLEIS, The Wong and Law Emotional Intelligence Scale; Emotional Traits: QET, Questionnaire of Emotional Traits; Resilience: RSCA, Resilience Scale for Chinese Adolescents, *p < 0.05. ***p < 0.001.

### Play and resilience: Serial mediation model

**Figure 1:**
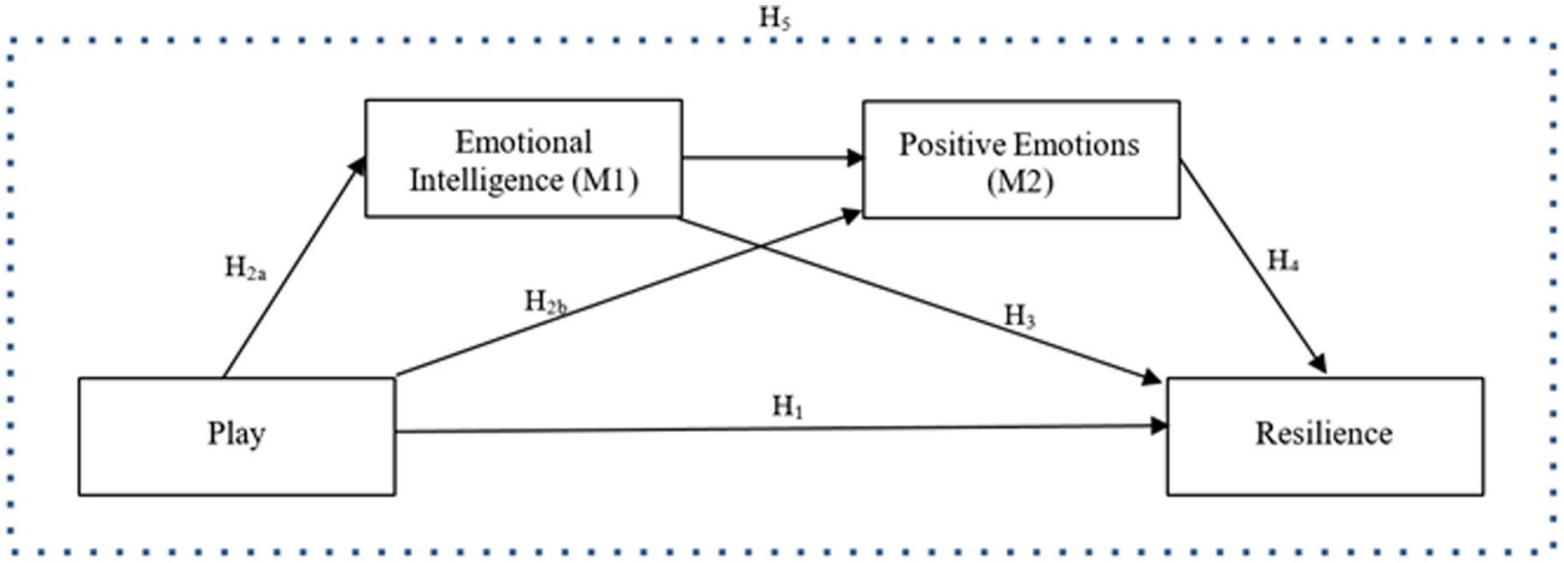
Hypothesized research model.

Sequential mediation analysis was used to investigate hypotheses 1 to 5. The hypothesized research model, which states that emotional intelligence (M1) and emotional traits (M2) will function as serial mediators in the relationship between play and resilience, is displayed in Figure 1. The model was implemented using the PROCESS macro for SPSS, and the indirect effects were determined by applying two mediators on the basis of 5,000 bootstrap samples in generating 95% bias-corrected bootstrap confidence intervals. The results of the PROCESS analyses are tabulated in Table 7. Van Jaarsveld et al. (2010) state that the advantage of this procedure enables isolation of each mediator’s indirect effect: emotional intelligence (Hypothesis 3) and emotional traits (Hypothesis 4). Additionally, this approach allows the investigation of “the indirect effect passing through both of these mediators in a series” (Hypothesis 5) (Van Jaarsveld et al., 2010, p. 1496).

**Figure 2:**
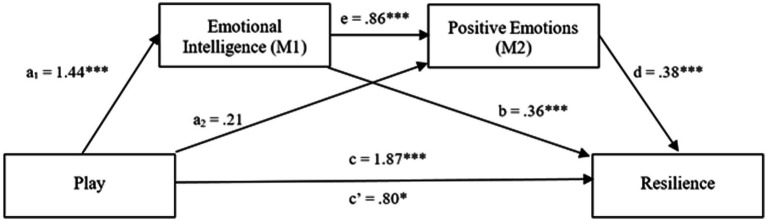
The serial mediation model. The serial mediation analysis for the relationship between play and resilience through emotional intelligence and emotional traits. c, total effect of play; c', direct effect of play with mediators controlled. Unstandardized regression coefficients are reported in this figure. *p < 0.05, ***p < 0.001.

The results of the serial mediation analyses are summarized in Figure 2. Confirming Hypothesis 1, the result showed a total effect of emerging adults’ play experiences on resilience, *B* = 1.87, *p* < 0.001. When the mediators were included in the analysis, this coefficient was reduced but was still statistically significant (direct effect, c’), *B* = 0.80, *p* = 0.02. Play was also found to be a positive predictor of emotional intelligence, *B* = 1.44, *p* < 0.001, but not emotional traits (i.e., positive emotion), *B* = 0.21, *p* = 0.74. Hypothesis 2 was partially supported. The results are tabulated in Table 6.

**Table 6:** Summary of multiple regression analyses for the serial mediation model.

Variable	Model 1					Model 2					Model 3				
	*B*	95% CI	*SE B*	*β*	*t*	*B*	95% CI	*SE B*	*β*	*t*	*B*	95% CI	*SE B*	*β*	*t*
Constant	39.42	[31.79, 47.04]	3.85		10.23^***^	31.56	[13.86, 49.27]	8.95		3.53^***^	24.02	[14.17, 33.87]	4.98		4.83^***^
Play	1.44	[0.78, 2.10]	0.33	0.36	4.31^***^	0.21	[−1.01, 1.42]	0.62	0.03	0.34	0.80	[0.15, 1.45]	0.33	0.15	2.44^*^
Emotional intelligence						0.86	[0.56, 1.16]	0.15	0.47	5.64^***^	0.36	[0.18, 0.54]	0.09	0.27	3.96^***^
Emotional traits											0.38	[0.29, 0.48]	0.05	0.54	8.19^***^
	*R*^2^ = 0.13, *F*_(1, 129)_ = 18.61, *p* < 0.001					*R*^2^ = 0.23, *F*_(2, 128)_ = 19.04, *p* < 0.001					*R*^2^ = 0.58, *F*_(3, 127)_ = 58.96, *p* < 0.001				

*N* = 131. Model 1: Predictor variable: play; outcome variable: emotional traits. Model 2: Predictor variables: play and emotional intelligence; outcome variable: emotional traits. Model 3: Predictor variables: play, emotional traits, and emotional intelligence; outcome variable: resilience. *p < 0.05. ***p < 0.001.

Table 7 shows the indirect effects and their associated 95% CIs. As shown in the table, the analysis of the indirect mediation by bootstrapping found that the resulting data supported the significance of Path 1 (Play ➔ Emotional Intelligence ➔ Resilience; *B* = 0.51, *SE* = 0.19, 95% CI [0.18, 0.93]), confirming Hypothesis 3, and Path 3 (Play ➔ Emotional Intelligence ➔ Emotional Traits ➔ Resilience; *B* = 0.47, *SE* = 0.17, 95% CI [0.19, 0.85]), confirming Hypothesis 5. However, Path 2 ([Play ➔ Emotional Traits ➔ Resilience; *B* = 0.08, *SE* = 0.28, 95% CI [−0.43, 0.67]) was non-significant. Hypothesis 4 was rejected.

**Table 7:** Bootstrapping indirect effects and 95% confidence interval (CI) for the sequential mediational model.

Pathway	Effect	Boot SE	95% CI	
			Lower	Upper
Play → Emotional intelligence → Resilience	0.51	0.19	0.18	0.93
Play → Emotional traits → Resilience	0.08	0.28	−0.43	0.67
Play → Emotional intelligence → Emotional traits → Resilience	0.47	0.17	0.19	0.85
Total	1.07	0.45	0.23	2.00

Confidence interval = CI; 5,000 bootstrap samples with 95% CI.

The results revealed a significant indirect effect of play on resilience through emotional intelligence and emotional traits (*b* = 0.47, *t* = 2.81), thereby supporting Hypothesis 5. Furthermore, the direct effect of play on resilience in presence of the mediators was also found to be significant (*b* = 0.80, *p* < 0.02). Hence, there was partial serial mediation of emotional intelligence and emotional traits on the relationship between play and resilience. The summary of the mediating effects is presented in Table 7. Consistent with previous research, play was related to high levels of resilience; however, as predicted, this relationship was mediated by emotional intelligence. These findings both support and extend prior research on emotional intelligence and resilience. Play appeared to positively affect resilience by enhancing emotional intelligence and positive emotions, which, in turn, predicted resilience.

## Discussion

Given the nature of the quantitative data, we conducted a serial mediation analysis and found that emotional intelligence and emotional traits mediated the positive relationship between play and resilience. The results convincingly demonstrated that play is an important predictor of emotional intelligence and resilience. These findings also addressed various concerns about play (i.e., types of play, playtime, and playmates).

Duffy et al. (2019) found an increased rate of anxiety, mood symptoms, and suicidal ideation among college students in the United States from 2007 to 2018. Similar results have been found in studies around the world including Hong Kong and the United Kingdom (e.g., Geulayov et al., 2016; Fong et al., 2022). This suggests that effective interventions for emerging adults are sorely needed at this time. The present study addressed this concern by examining the mediating roles of emotional intelligence and positive emotions in the relationship between play and resilience. In support of Hypothesis 1, this study found that emerging adults’ play experiences positively predicted resilience. This aligned with previous findings showing that leisure activities as a form of play are conducive to resilience (Denovan and Macaskill, 2017). Play serves as an oasis to recharge physically, psychologically, and/or emotionally, which contributes to the resilience of young people (Gilligan, 2000). Next, the current study found that play experiences positively predicted emotional intelligence (supporting Hypothesis 2, H2a), suggesting that emerging adults who reported more positive play experience tended to develop greater emotional intelligence. This finding is supported by Goleman (2006), who posited that play is a way to develop emotional intelligence.

However, this study did not find a significant association between play and positive emotions (not supporting Hypothesis 2, H2b). There are two plausible explanations for this. First, the content of play may involve both positive (e.g., winning, joy, fun, and happiness) and negative themes (e.g., losing, pain, sadness, anger, and frustration). For example, a player may channel their aggression through sports. The act of playing allows people to express and transform unpleasant or horrifying feelings, such as pain, loss, or death (Winnicott, 2017). Play enables people to cognitively resolve conflicting emotions and relieve negative emotional states (Mainemelis and Ronson, 2006). The cognitive element of play may change and generally result in some form of positive effect but only after some time. Second, this study was conducted during the COVID-19 pandemic; thus, individuals may have experienced high anxiety and negative emotions, which could have lowered the level of positive emotions. Another study showed that the COVID-19 pandemic created a dynamic interplay of emotions and an increase in negative emotions, particularly anger (Kim et al., 2022). Therefore, further studies are considered.

Our findings showed that emotional intelligence had a mediating effect between play and resilience in emerging adults (supporting Hypothesis 3), showing that play contributed to the development of emotional intelligence, thereby promoting resilience. The instinct to play is ingrained in us throughout our lives (Carroll, 2009) and influences the development of emotional intelligence skills. Individuals with a higher level of emotional intelligence display a more stable sense of resilience throughout life (Shuo et al., 2022). Our findings aligned with previous research and confirmed that emotional intelligence has a significant effect on resilience (Tugade and Fredrickson, 2004).

It is surprising that, in our study, positive emotions did not play an intermediary role in the relationship between play experiences and resilience in emerging adults (thus, Hypothesis 4 was not supported). In this respect, our findings differed from previous studies. For example, Newman et al. (2014) reported that individuals experience positive emotions through meaningful leisure engagement, which promotes stress-coping abilities and resilience. As discussed in H2b, it is necessary to conduct further research to examine the mediating effect of emotional traits, both positive and negative.

The serial mediation analysis showed that emotional intelligence and positive emotions operate as serial mediators between emerging adults’ play experiences and resilience (supporting Hypothesis 5). This finding is in line with previous studies. Emerging adults develop emotional intelligence through play. Individuals who have higher emotional intelligence cope better with stressful encounters and produce more adaptive responses (Salovey et al., 1999; Lea et al., 2019). Resilient people are often described as being emotionally intelligent (Salovey et al., 1999) and appear to use positive emotions in their favor (Tugade and Fredrickson, 2002). Fredrickson (1998, 2001) claimed that positive emotions have a complementary effect, widening the array of the thoughts and actions that come to mind.

To date, studies have rarely directly examined the mediated relationship between emerging adults’ play experiences and resilience. By examining the mediating roles of emotional intelligence and emotional traits in the relationship between play and resilience, this study extended the literature of play on psychological benefits in emerging adults. According to previous studies (e.g., Saklofske et al., 2007; Shikako-Thomas et al., 2013; Green, 2017), play was positively correlated with individuals’ experiences of pleasure and enjoyment, knowledge acquisition, development of new skills, emotional intelligence, emotional traits, and resilience.

The current study adds to the evidence that emerging adults’ playful experiences have a positive impact on their psychological development. The results indicated that emerging adults had higher emotional intelligence, more positive moods, and greater resilience when they engaged more in positive play experiences. Hence, promoting positive play experience results in improving emotional intelligence and enhancing positive emotions, which allows emerging adults to develop key abilities to rebound from stressful events. Therefore, it is essential that emerging adults engage in play.

### Implications for future research, professional practice, and education

Play is considered essential to optimize individual growth and development (Bayliss and Etchells, 2016). Our study found that play is an effective an effective means to enhance emotional intelligence, promote positive emotions, and build resilience. As shown in other studies (Armstrong et al., 2011; Magnano et al., 2016; Sarrionandia et al., 2018), emotional intelligence plays a significant role in resilience.

Research into factors that influence emerging adults’ psychological development is valuable in that it reveals ways in which it can be supported and highlight new directions to consider when developing interventions. First, play is seen as an effective means because of its impact on emotional intelligence and resilience which, in turn, affect emerging adults’ psychological development.

Second, the majority of previous studies focused on children and youth (e.g., Ginsburg et al., 2007; Yogman et al., 2018) or on electronic play (e.g., Müller and Bonnaire, 2021; TaeHyuk Keum and Hearns, 2022). In the present study, most emerging adults spent less than 7 h weekly on play/extracurricular activities (80%) and leisure reading (93%) in the last 12 months. This implies that few people currently engage in regular play/extracurricular activities and leisure reading. Indeed, around 60% of the individuals spent more than 7 h weekly playing video games. Even though exploring the negative impacts of electronic play are necessary, examining its appropriateness and effectiveness for emerging adults is of crucial importance.

Third, universities should make a variety of play activities available to their students. Regardless of age, play evokes enjoyment (Glenn and Knapp, 1987) and promotes intimacy in romantic relationships (Oring, 1984). Play can also serve as a tool for conflict resolution (Betcher, 1981; Glenn and Knapp, 1987; Baxter, 1992). Therefore, play is a particularly significant resource for promoting positive psychological development. In this study, 92.4% of the participants reported that they have playmates and 80.2% always played with friends. However, 7.6% of the participants reported that they have no playmates. Educators should not ignore the risk to this group of students.

### Limitations and recommendations for further research

This study had three shortcomings. Frist, everyone can freely choose to play. This study cannot answer which types of play are most effective. Individuals define play according to their subjective perspectives. Further investigation with regard to type, frequency, and duration of play to improve psychological development and mental health outcomes should be considered.

A second limitation of this study is gender bias within the sample as the majority of the participants were women (93.9%). This is reflected by its small sample size and the gender imbalance between the groups. Thus, the results may be unrepresentative of other genders (Ritchie, 2009). Due to sampling convenience and sampling scarcity, the researchers selected individuals if they had some sense about the definition of play (i.e., play knowledge and experience), despite the obvious fact that there were more women than men in the sample. This non-probability sampling was not intended to be representative of a population. The results are subject to auto-selection bias, and the findings might have limited generalizability or external validity. Further studies should investigate larger samples to reduce sampling error and increase external validity.

Third, play is age specific. Individuals at different ages and developmental stages should choose age-appropriate play. Human development encompasses changes in physical, behavioral, cognitive, social, and emotional characteristics over the course of a lifetime. The current study focused only on psychological development. Owing to time limitations and the study’s cross-sectional design, longitudinal data were excluded. In the future, longitudinal studies should evaluate other psychological variables (e.g., self-identity, intimate relationship, parenting styles, childhood play experiences) and investigate the impact of play at various developmental stages.

## Conclusion

This study found that emotional intelligence and emotional traits both significantly mediated the relationship between play and resilience. In emerging adults’ play experiences, play was a predictor of resilience, and this relationship was serially mediated by emotional intelligence and positive emotions. Given the value of play, emerging adults should enhance positive play experiences, particularly sporting activities, to maintain a healthy work-life balance and should give consideration to the meaningful role that play has in their lives.

## Data availability statement

The datasets presented in this study can be found in online repositories. The names of the repository/repositories and accession number(s) can be found at: https://osf.io/b2ae5/?view_only=e0352ab1943243dea95b5f2c0b260df4.

## Ethics statement

The studies involving human participants were reviewed and approved by Research Ethics Committee, Hong Kong Metropolitan University. The patients/participants provided their written informed consent to participate in this study.

## Author contributions

The author confirms being the sole contributor of this work and has approved it for publication.

## Conflict of interest

The author declares that the research was conducted in the absence of any commercial or financial relationships that could be construed as a potential conflict of interest.

## Publisher’s note

All claims expressed in this article are solely those of the authors and do not necessarily represent those of their affiliated organizations, or those of the publisher, the editors and the reviewers. Any product that may be evaluated in this article, or claim that may be made by its manufacturer, is not guaranteed or endorsed by the publisher.
